# Clinical and cardiometabolic factors associated with insulin resistance in an adult population from a high HIV‐burden setting in Zambia

**DOI:** 10.14814/phy2.71159

**Published:** 2026-11-10

**Authors:** Situmbeko Liweleya, Brian Halubanza, Hatwiko Hanzooma, Taonga Tembo, Lweendo Muchaili, Martin Chakulya, Lukundo Siame, Propheria C. Lwiindi, Luyando Mutelo, Joreen P. Povia, Freeman M. Chabala, Musalula Sinkala, Annet Kirabo, Sepiso K. Masenga, Benson M. Hamooya

**Affiliations:** ^1^ HAND Research Group, School of Medicine and Health Sciences Mulungushi University, Livingstone Campus Livingstone Zambia; ^2^ Department of Cardiovascular Science and Metabolic Diseases Livingstone Center for Prevention and Translational Science Livingstone Zambia; ^3^ School of Engineering and Technology Mulungushi University Kabwe Zambia; ^4^ Department of Health Economics Livingstone Center for Prevention and Translational Sciences Livingstone Zambia; ^5^ The Institute of Basic and Biomedical Sciences Levy Mwanawasa Medical University Lusaka Zambia; ^6^ Division of Computational Biology, Department of Integrative Biomedical Sciences, Faculty of Health Sciences University of Cape Town Cape Town South Africa; ^7^ Institute of Infectious Diseases and Molecular Medicine, Faculty of Health Sciences University of Cape Town Cape Town South Africa; ^8^ Department of Biomedical Sciences, School of Health Sciences University of Zambia Lusaka Zambia; ^9^ Division of Genetics and Clinical Pharmacology, Department of Medicine Vanderbilt University Medical Center Nashville Tennessee USA; ^10^ Vanderbilt Center for Immunobiology Nashville Tennessee USA; ^11^ Vanderbilt Institute for Infection, Immunology and Inflammation Nashville Tennessee USA; ^12^ Vanderbilt Institute for Global Health Nashville Tennessee USA; ^13^ Department of Molecular Physiology and Biophysics Vanderbilt University Nashville Tennessee USA

**Keywords:** cardiometabolic, central obesity, HIV, hypertension, insulin resistance, metabolic syndrome

## Abstract

Insulin resistance (IR) is a key pathophysiological link between metabolic syndrome and type 2 diabetes. Among people living with HIV (PLWH), both antiretroviral therapy (ART) and chronic immune activation may potentiate IR. This study aimed to identify clinical and cardiometabolic correlates of IR. We conducted a cross‐sectional study among 233 adults (142 PLWH and 91 people without HIV) attending routine medical checkups at Livingstone University Teaching Hospital, Zambia. Fasting glucose and insulin were used to calculate HOMA2‐IR, with values >1.9 defining IR. Anthropometry, blood pressure, lipid parameters, hs‐CRP, renal function, and echocardiographic measures were assessed. Multivariable logistic regression estimated adjusted odds ratios (aORs) and 95% confidence intervals, with HIV status retained a priori. Model selection emphasized parsimony, while multicollinearity was assessed using Spearman correlations and variance inflation factors (VIFs). Highly correlated predictors were not entered simultaneously. Model discrimination and calibration were assessed using the AUC and Hosmer‐Lemeshow goodness‐of‐fit test, respectively. IR was present in 37 of 233 participants (15.9%). Compared with participants without IR, those with IR had higher BMI (28.4 vs. 24.45 kg/m^2^, *p* = 0.0002), waist circumference (99.7 vs. 82.5 cm, *p* < 0.0001), total cholesterol (4.87 vs. 4.31 mmol/L, *p* = 0.046), triglycerides (0.950 vs. 0.755 mmol/L, *p* = 0.0068), and VLDL cholesterol (0.400 vs. 0.300 mmol/L, *p* = 0.005), as well as higher hs‐CRP concentrations (*p* = 0.0008). IVSD and LVPWD were also greater among participants with IR (*p* = 0.002 and *p* = 0.016, respectively). In the parsimonious multivariable model, waist circumference was independently associated with higher odds of IR (aOR 1.045 per cm; 95% CI: 1.012–1.080; *p* = 0.008), while HIV status, hypertension, triglycerides, and IVSD were not independently associated with IR. The model demonstrated moderate discrimination (AUC = 0.767; 95% CI: 0.676–0.858) with no evidence of significant lack of calibration (Hosmer–Lemeshow *p* = 0.164). IR was present in approximately one in six adults in this high HIV‐burden setting and was characterized by greater adiposity, dyslipidemia, inflammation, and cardiac structural differences. After adjustment, WC was the only independent correlate of IR, while HIV status was not independently associated. These findings underscore the importance of incorporating adiposity and broader cardiometabolic assessment into routine care, including HIV care, and support prospective studies to clarify the temporal and mechanistic relationships between these factors.

## 1 INTRODUCTION

Insulin resistance (IR) is a fundamental defect in the pathophysiology of type 2 diabetes and a major driver of atherosclerotic cardiovascular disease (CVD) and metabolic syndrome (Kosmas et al., 2023). It is characterized by a diminished biological response of target tissues (liver, muscle, adipose) to normal circulating levels of insulin, leading to compensatory hyperinsulinemia (Freeman et al., 2025).

The global HIV epidemic has evolved into a chronic disease model, with life expectancy for people living with HIV (PLWH) on antiretroviral therapy (ART) approaching that of the general population (Pan et al., 2024; Wing, 2017). However, this success is tempered by a rising burden of non‐communicable diseases (NCDs), particularly metabolic and cardiovascular conditions (Kibret et al., 2023). PLWH face a unique confluence of risks for IR: some ART regimens are associated with metabolic disturbances; chronic, low‐grade immune activation persists even with viral suppression; and lifestyle factors may compound these risks (Mokoena et al., 2025; Wang et al., 2025).

In sub‐Saharan Africa (SSA), which bears the greatest global burden of HIV, the intersection of HIV, IR, and CVD is under‐studied yet critically important (Bain & Gwain, 2019). Health systems are often geared towards infectious disease management, with less capacity for integrated NCD care. Furthermore, population‐specific data on the cardiometabolic correlates of IR in PLWH are scarce.

This study aimed to identify the clinical, metabolic, and cardiovascular factors associated with IR in a cohort from Zambia, a high HIV‐burden setting. By including both PLWH and people living without HIV (PLWoH), we sought to delineate the specific determinants of IR and inform targeted prevention strategies within HIV care programs.

## 2 METHODS

### 2.1 Study design and participants

This cross‐sectional study recruited 233 adults (142 [60.9%] PLWH, 91 [39.1%] PLWoH) from a cohort of individuals who attended routine medical checkups at Livingstone University Teaching Hospital (LUTH), a tertiary hospital in Livingstone, Southern Province, Zambia. The study was approved by the Mulungushi University Ethics Review Committee (SMHS‐MU1‐2025‐39). Written informed consent was obtained from all participants before enrolment. The study was conducted in accordance with the principles of the Declaration of Helsinki.

### 2.2 Eligibility

Adults aged 18 years and older were enrolled. PLWH had a confirmed diagnosis, were receiving stable ART for at least 6 months, and had achieved viral suppression (viral load <50 copies/mL). PLWoH (controls) were recruited from general medicine clinics. Exclusion criteria included known diabetes, pregnancy, severe kidney or liver disease, active opportunistic infections, or other major comorbid conditions, apart from obesity that could confound inflammatory and metabolic measurements.

### 2.3 Data collection

#### 2.3.1 Demographic and clinical data

Standardized questionnaires administered via Research Electronic Data Capture (REDCap) collected information on age, sex, employment, marital status, and medical history (including history of tuberculosis).

#### 2.3.2 Anthropometric measurements

Height and weight were measured using a calibrated stadiometer. Body mass index (BMI) was calculated as weight/height^2^ (kg/m^2^). Waist circumference was measured at the midpoint between the lower rib margin and the iliac crest.

#### 2.3.3 Clinical and laboratory measurements

Fasting venous blood samples were obtained. Glucose, insulin, and a full lipid panel (total cholesterol, LDL‐c, HDL‐c, triglycerides) were analyzed. Very low‐density lipoprotein (VLDL) and non‐HDL cholesterol were calculated. High‐sensitivity C‐reactive protein (hs‐CRP) was measured using ELISA. Serum creatinine and uric acid were measured, and the estimated glomerular filtration rate (eGFR) was derived using the CKD‐EPI equation (2021).

Fasting venous blood samples were collected after an overnight fast of at least 8–10 h. Blood samples for biochemical analyses were collected into serum separator tubes and lithium‐heparin tubes. Serum separator tubes were allowed to clot for 30 min at room temperature (20°C–25°C), after which blood samples were centrifuged at approximately 1500–2000 × g for 10–15 min at room temperature. Serum or lithium‐heparin plasma was subsequently separated from cellular components and stored at 2°C–8°C until analysis. The specimen type, processing interval, and storage conditions were standardized across participants to minimize pre‐analytical variation.

Glucose was measured enzymatically on a Pentra C200 clinical chemistry analyzer (HORIBA Medical, Montpellier, France). The laboratory documentation identifies the assay as enzymatic and indicates hexokinase or glucose‐oxidase methodology; the glucose‐oxidase method was used. Results were reported in mmol/L.

Total cholesterol was measured on the Pentra C200 analyzer (HORIBA Medical, Montpellier, France) using the cholesterol oxidase‐phenol aminophenazone (CHOD‐PAP) enzymatic colorimetric method, with results reported in mmol/L.

HDL‐C was measured on the Pentra C200 analyzer (HORIBA Medical, Montpellier, France) using a direct homogeneous enzymatic assay, and results were reported in mmol/L.

Triglycerides were measured on the Pentra C200 analyzer (HORIBA Medical, Montpellier, France) using the glycerol‐3‐phosphate oxidase‐phenol aminophenazone (GPO‐PAP) enzymatic method, with results reported in mmol/L.

LDL‐C concentrations were estimated using the Friedewald equation, based on measured total cholesterol, HDL‐C, and triglyceride concentrations. The equation was applied as follows: LDL‐C = total cholesterol‐HDL‐C‐(triglycerides/2.2), with all lipid concentrations expressed in mmol/L. The calculation was restricted to fasting samples with triglyceride levels below 4.5 mmol/L, in accordance with the conventional limits of the Friedewald method.

High‐sensitivity C‐reactive protein was quantified using a commercially available pre‐coated sandwich ELISA kit (manufacturer; Elabscience, catalog number; E‐EL‐H5134, country, USA), according to the manufacturer's instructions. The assay was validated for serum and EDTA plasma. Following completion of the enzyme‐substrate reaction and addition of stop solution, absorbance was measured at 450 nm using a BioTek ELx800 microplate reader (BioTek Instruments, Inc., Winooski, VT, USA). Microplate washing was performed using a BioTek ELx50 Microplate Strip Washer. Analyte concentrations were determined from plate‐specific standard curves generated from the optical‐density measurements. Assay validity was assessed using duplicate concordance, the working range of the standard curve, and the manufacturer's specified performance criteria. The analytical range and intra‐assay and inter‐assay coefficients of variation should be inserted from the kit manufacturer's documentation. The uploaded laboratory document reports the ELISA results in pg/mL.

Serum uric acid was measured using the ABX Pentra Uric Acid CP reagent (HORIBA ABX SAS, Montpellier, France; catalog/reference no. A11A01670) on a Pentra C200 clinical chemistry analyzer (HORIBA Medical, Montpellier, France). Plasma collected in lithium‐heparin tubes was used for analysis. The assay is an enzymatic colorimetric method based on the uricase‐peroxidase (Trinder) reaction, in which uric acid is oxidized by uricase to allantoin, carbon dioxide, and hydrogen peroxide; the hydrogen peroxide subsequently reacts with 4‐aminoantipyrine and EHSPT in the presence of peroxidase to form a chromogenic quinoneimine product, the intensity of which is proportional to the uric acid concentration.

Serum creatinine was measured using the ABX Pentra Enzymatic Creatinine CP reagent (HORIBA ABX SAS, Montpellier, France; Ref. A11A01907) on a Pentra C200 automated clinical chemistry analyzer (HORIBA Medical, Montpellier, France). Lithium‐heparin plasma was used for analysis. The assay employs a multi‐step enzymatic colorimetric method with a photometric endpoint. Briefly, creatinine is enzymatically converted to creatine by creatinine amidohydrolase, followed by conversion of creatine to sarcosine and urea by creatine amidinohydrolase. Sarcosine is subsequently oxidized by sarcosine oxidase, generating hydrogen peroxide, which reacts with 4‐aminoantipyrine and N‐ethyl‐N‐sulfopropyl‐m‐toluidine in a peroxidase‐mediated reaction to form a quinoneimine chromogen measured at 545 nm. Creatinine concentrations were reported in μmol/L.

#### 2.3.4 Blood pressure and hypertension definition

Blood pressure (BP) was measured three times using an Omron HEM‐7120 device, and the average was used. Hypertension was defined per JNC 8 criteria: SBP ≥140 mmHg and/or DBP ≥90 mmHg, or current use of antihypertensive medication.

#### 2.3.5 Echocardiography

Comprehensive transthoracic echocardiography was performed. Left ventricular structural parameters, including interventricular septal diameter in diastole (IVSD) and left ventricular posterior wall diameter in diastole (LVPWD), were obtained per American Society of Echocardiography guidelines (Mitchell et al., 2019). *Hypertensive heart disease* was defined as structural abnormalities of the left side of the heart, including the atrium and the presence of left ventricular hypertrophy (LVH) in a hypertensive individual (Nemtsova et al., 2023).

### 2.4 Cardiovascular risk and kidney function estimates

Estimated glomerular filtration rate (eGFR) was derived from serum creatinine via the CKD‐EPI (2021). equation, adjusted for age and sex (Roy et al., 2025).

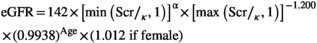

where:

Scr = standardized serum creatinine in mg/dL.

*κ* = 0.7 (females) or 0.9 (males).

*α* = −0.241 (female) or −0.302 (male).

min(Scr/*κ*, 1) is the minimum of Scr/*κ* or 1.0.

max(Scr/*κ*, 1) is the maximum of Scr/*κ* or 1.0.

Age (years).

*Insulin resistance* refers to a metabolic condition in which body tissues, primarily the liver, muscle, and adipose tissue, exhibit a reduced biological response to normal circulating levels of insulin. Clinically, IR is often assessed using surrogate indices such as the HOMA2 model, which estimate insulin sensitivity based on fasting glucose and insulin concentrations (Aliyu et al., 2025; Cersosimo et al., 2014; Ranganath & Varghese, 2024). To accomplish this, we applied a predefined threshold of HOMA2‐IR 1.9, which is consistent with published epidemiological and metabolic research identifying early IR (Aliyu et al., 2025; Liweleya et al., 2026). This value is indicative of clinically meaningful IR (Bermúdez et al., 2014). Values above the 1.9 cutoff were coded as “1” (elevated IR), and values at or below the cutoff were coded as “0” (Gayoso‐Diz et al., 2013). The population‐specific thresholds for HOMA2‐IR have not been established for this population; a threshold of >1.9 was adopted operationally to classify IR, informed by previously published HOMA‐based thresholds. This threshold should not be interpreted as a population‐specific diagnostic cutoff.

### 2.5 Data analysis plan

Multivariable logistic regression was used to estimate adjusted odds ratios (aORs) and 95% confidence intervals for factors associated with IR. HIV status was retained as an a priori covariate because of the study objective. Candidate predictors were selected based on clinical relevance and findings from the descriptive analyses, with attention to model parsimony given the number of insulin‐resistance events. Multicollinearity was assessed using pairwise Spearman correlation coefficients and variance inflation factors (VIFs); a VIF ≥5 was considered indicative of potentially important collinearity. Highly correlated predictors were not entered simultaneously into the primary model. Waist circumference was retained as the principal measure of adiposity, triglycerides as the measured lipid marker, and IVSD as the principal echocardiographic parameter. Alternative correlated variables were evaluated in sensitivity analyses. Model discrimination and calibration were assessed using the area under the receiver operating characteristic curve and the Hosmer‐Lemeshow goodness‐of‐fit test, respectively.

## 3 RESULTS

The prevalence of IR (HOMA2‐IR >1.9) in the cohort of 233 participants was 15.9% (37/233). The general characteristics of the cohort, stratified by IR status, are presented in Table 1. Hypertension was more prevalent among participants with IR (28.6% vs. 11.2%, *p* = 0.0013). The median age in people with IR was higher 54 (45.66, 55.58) than those without IR 47 (45.46, 49.31). There were no significant differences in the distribution of sex, history of TB, microalbuminuria, hypertensive heart disease, PAD, or HIV status between the IR and non‐IR groups (Table 1).

**TABLE 1:** General characteristics and distribution of insulin resistance.

Variables	Frequency (%)/median (IQR)/mean (SD)	Insulin resistance no, *n* (%) 196 (84.1)	Insulin resistance yes, *n* (%) 37 (15.9)	*p*‐value
Age	49 (40, 59)	47 (45.46, 49.31)	54 (45.66, 55.58)	0.074
Sex, *n* = 233				
Male	79 (34.0)	69 (87.3)	10 (12.7)	0.335
Female	154 (66.0)	127 (82.5)	27 (17.5)	
Hypertension, *n* = 233				
Hypertensive	63 (27.0)	45 (71.4)	18 (28.6)	**0.0013**
Normotensive	170 (73.0)	151 (88.8)	19 (11.2)	
History of TB				
No	163 (86.7)	138 (84.7)	25 (15.3)	0.662
Yes	25 (13.3)	22 (88.0)	3 (12.0)	
Microalbuminuria				
No	124 (67.0)	104 (83.9)	20 (16.1)	0.590
Yes	61 (33.0)	53 (86.9)	8 (13.1)	0.137
Hypertensive heart disease				
No	194 (90.0)	165 (85.1)	29 (14.9)	
Yes	22 (10.0)	16 (72.7)		
PAD				
No	184 (84.0)	155 (84.2)	29 (15.8)	0.838
Yes	35 (16.0)	29 (82.9)	6 (17.1)	
HIV				
No	91 (39.0)	73 (80.2)	18 (19.8)	0.192
Yes	142 (60.9)	123 (86.6)	19 (13.4)	

*Note*: Bold *p*‐value indicates statistical significance.

### 3.1 Clinical and cardiometabolic characteristics according to insulin resistance status

Participants were stratified according to IR status using the HOMA2‐IR cutoff of >1.9. Of the 233 participants, 37 (15.9%) were classified as having IR, while 196 (84.1%) were classified as not having insulin resistance. Compared with participants without IR, those with IR had significantly greater adiposity, reflected by higher BMI (28.4 vs. 24.45 kg/m^2^, *p* = 0.0002) and waist circumference (99.7 vs. 82.5 cm, *p* < 0.0001). Participants with IR also had higher total cholesterol (4.87 vs. 4.31 mmol/L, *p* = 0.046), triglycerides (0.950 vs. 0.755 mmol/L, *p* = 0.0068), and VLDL cholesterol (0.400 vs. 0.300 mmol/L, *p* = 0.005). hs‐CRP concentrations were higher among participants with IR (*p* = 0.0008). Echocardiographic assessment demonstrated greater interventricular septal diameter (IVSD; *p* = 0.002) and left ventricular posterior wall diameter (LVPWD; *p* = 0.016) in participants with IR. No significant differences were observed for age, LDL cholesterol, HDL cholesterol, LDL/HDL ratio, cholesterol/HDL ratio, creatinine, uric acid, eGFR, SSBP delta, or SST2 (all *p* > 0.05) (Figure 1).

**FIGURE 1:**
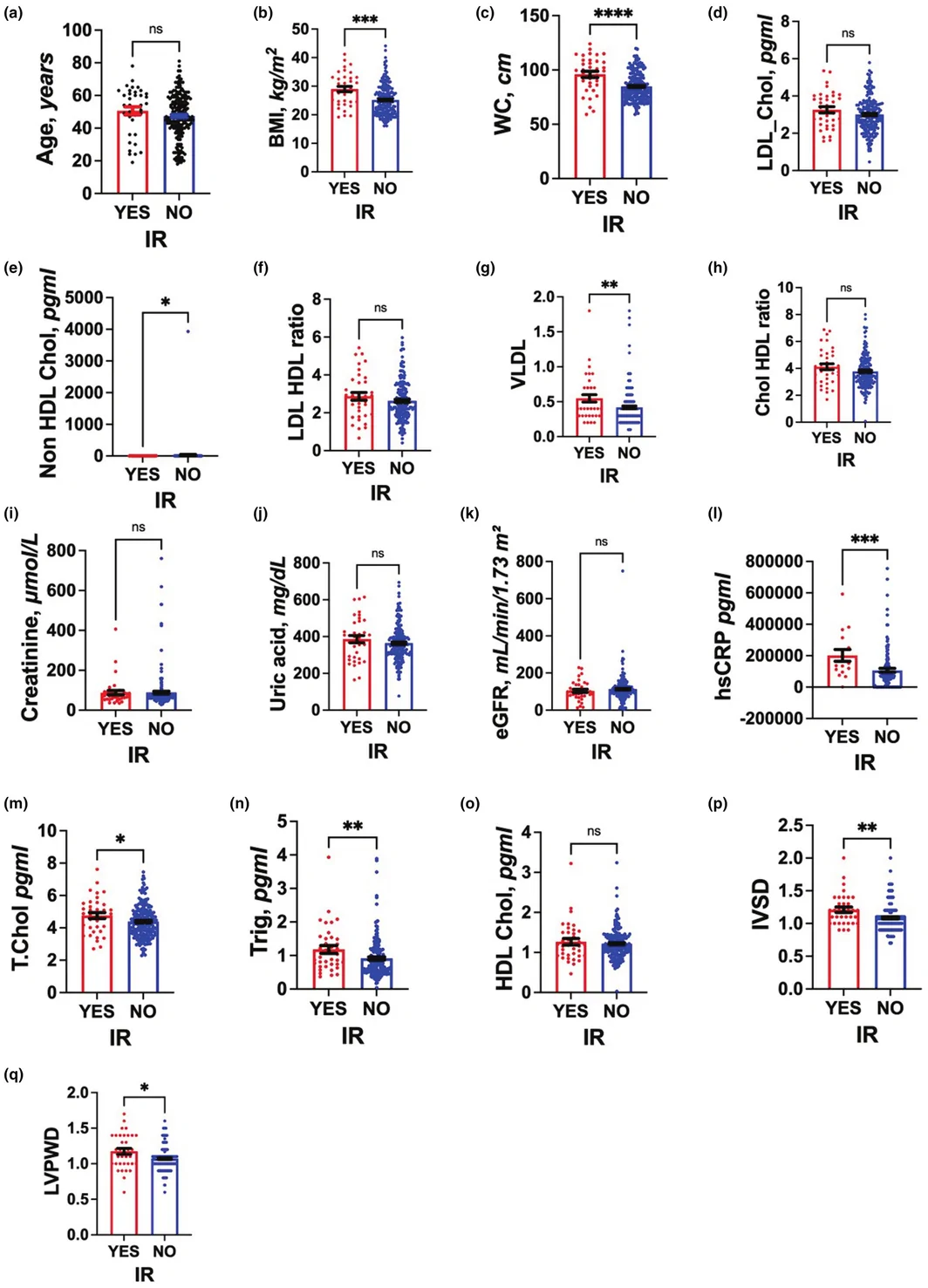
Characteristics of IR by anthropometric, metabolic, renal, cardiovascular and hemodynamic markers between people with and without IR respectively: (a) Age, years, (b) BMI, kg/m^2^, (c) Waist circumference, cm, (d) LDL cholesterol, pgml, (e) non‐HDL cholesterol, pgml, (f) LDL‐HDL ratio, (g) VLDL, pgml, (h) Cholesterol‐HDL ratio, (i) Creatinine, μmol/L, (j) Uric acid, mg/dl, (k) eGFR, mL/min/1.73m^2^, (l) hs_CRP, pgml, (m) Total cholesterol, pgml, (n) Triglycerides, pgml, (o) HDL cholesterol, pgml, (p) IVSD, (q) LVPWD. BMI, Body mass index; eGFR, Estimated glomerular filtration Rate; HDL_cholesterol, High‐Density lipoprotein cholesterol; hs_CRP, High‐sensitivity C‐Reactive protein; IVSD, Interventricular septal diameter in diastole; LDL_cholesterol, Low‐Density lipoprotein cholesterol; LVPWD, Left ventricular posterior wall diameter in diastole; VLDL, very low‐density lipoprotein; WC, Waist circumference, Statistical significance of differences between participants with and without insulin resistance: ns, not statistically significant; * *p* < 0.05; ** *p* < 0.01; *** *p* < 0.001; ****p < 0.0001.

### 3.2 Multicollinearity diagnostics for candidate predictors of IR

Multicollinearity was generally low among candidate predictors, except for VLDL (VIF = 39.98) and triglycerides (VIF = 39.73), which demonstrated severe collinearity. IVSD and LVPWd showed moderate collinearity (VIF = 4.36 and 4.52, respectively). These findings informed model refinement, with highly collinear predictors not entered simultaneously in the primary multivariable model (see Table 2).

**TABLE 2:** Multicollinearity diagnostics for candidate predictors.

Predictor	VIF
HIV	1.21
Hypertension	1.52
Waist circumference	1.59
IVSD	4.36
LVPWd	4.52
VLDL	**39.98**
Triglycerides	**39.73**
Non‐HDL cholesterol	1.07
hs‐CRP	1.24

*Note*: Bold *p*‐value indicates statistical significance.

### 3.3 Multivariable logistic regression model diagnostics for IR

Model diagnostics demonstrated substantial improvement following model simplification. The original model included nine predictor parameters among 106 complete cases with 15 insulin‐resistance events, corresponding to 1.7 events per parameter and a maximum VIF of 39.98, indicating substantial model complexity and multicollinearity. Although the original model showed high apparent discrimination (AUC = 0.938), the significant Hosmer–Lemeshow test (*p* = 0.037) indicated evidence of poor calibration. The recommended primary model included five predictor parameters among 185 complete cases with 33 insulin‐resistance events, increasing the events‐per‐parameter ratio to 6.6 and reducing the maximum VIF to 1.32. The revised model demonstrated moderate discriminatory ability (AUC = 0.767) and no evidence of significant lack of calibration (Hosmer–Lemeshow *p* = 0.164), supporting a more parsimonious and stable model specification (see Table 3).

**TABLE 3:** Multivariable logistic regression model diagnostics.

Diagnostic	Original model	Recommended primary model
Total participants available	233	233
Complete cases	106	185
IR events	15	33
Number of predictor parameters	9	5
Events per parameter	1.7	6.6
Maximum VIF	39.98	1.32
AUC	0.938*	0.767
Hosmer–Lemeshow *p*	0.037*	0.164

* *p* < 0.05.

### 3.4 ROC curve for the multivariate model for predicting insulin resistance

ROC curve demonstrating the discriminatory performance of the multivariable logistic regression model for insulin resistance. The model included 185 participants, including 33 participants with insulin resistance. The area under the ROC curve (AUC) was 0.767 (95% CI: 0.676–0.858), indicating moderate discriminatory ability. The diagonal dashed line represents chance discrimination (AUC = 0.50). Model calibration was assessed using the Hosmer–Lemeshow goodness‐of‐fit test (*χ* ^2^ = 10.03, df = 8, *p* = 0.164) (see Figure 2).

**FIGURE 2:**
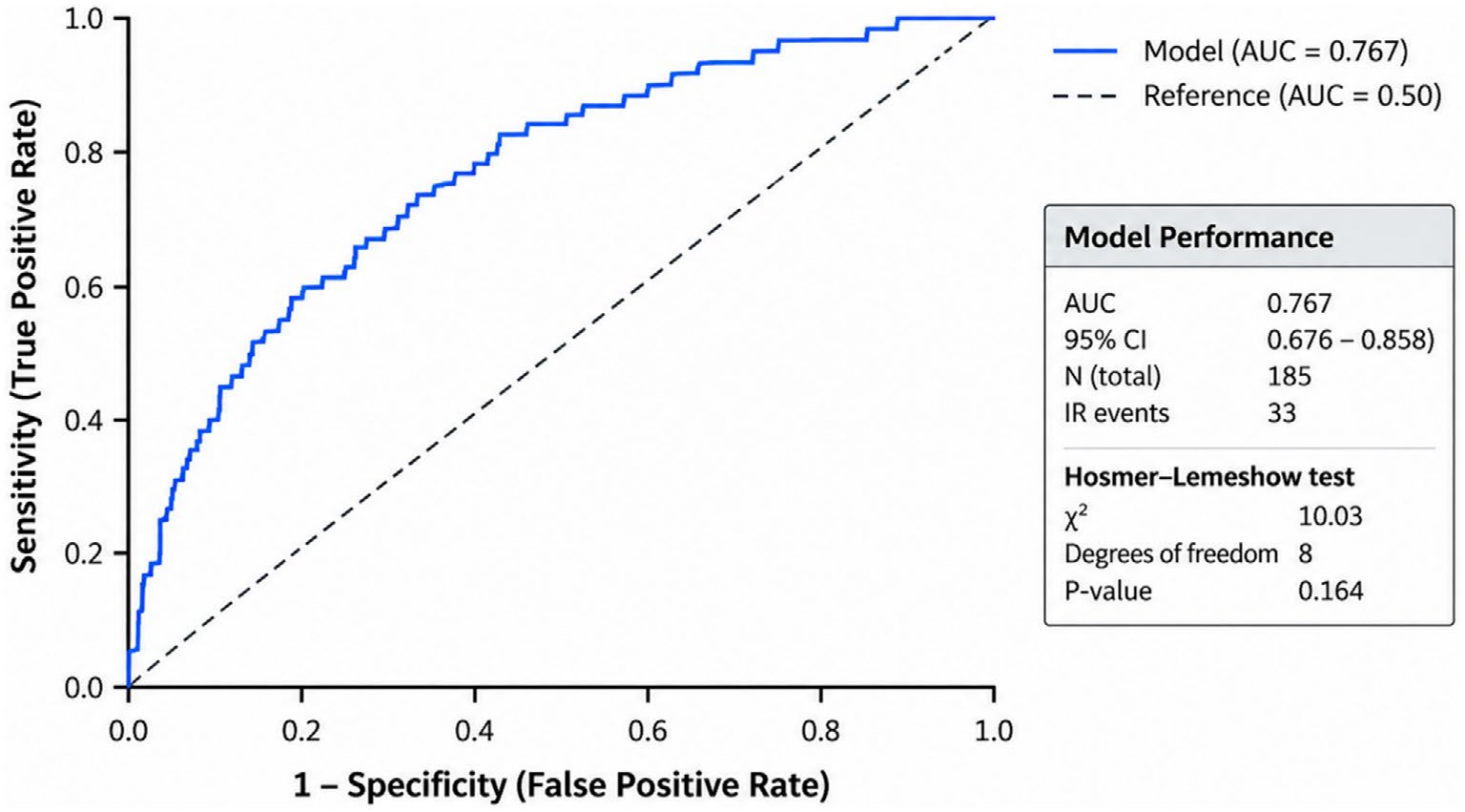
ROC curve for the multivariate model for predicting insulin resistance. Variance inflation factors (VIFs) for all predictors were low (<2), suggesting no evidence of problematic multicollinearity. The model demonstrated good calibration (Hosmer–Lemeshow *p* = 0.164) and moderate discriminatory ability (AUC = 0.767).

### 3.5 Multivariate analysis

In the multivariable logistic regression model (*n* = 185; 33 insulin‐resistance events), waist circumference was independently associated with higher odds of insulin resistance (aOR 1.045 per cm increase; 95% CI: 1.012–1.080; *p* = 0.008). HIV status (aOR 0.66; 95% CI: 0.28–1.59; *p* = 0.357), hypertension (aOR 2.02; 95% CI: 0.83–4.92; *p* = 0.123), triglycerides (aOR 1.46; 95% CI: 0.79–2.71; *p* = 0.228), and interventricular septal diameter (IVSD) (aOR 4.34; 95% CI: 0.72–26.04; *p* = 0.109) were not independently associated with insulin resistance after adjustment (see Figure 3).

**FIGURE 3:**
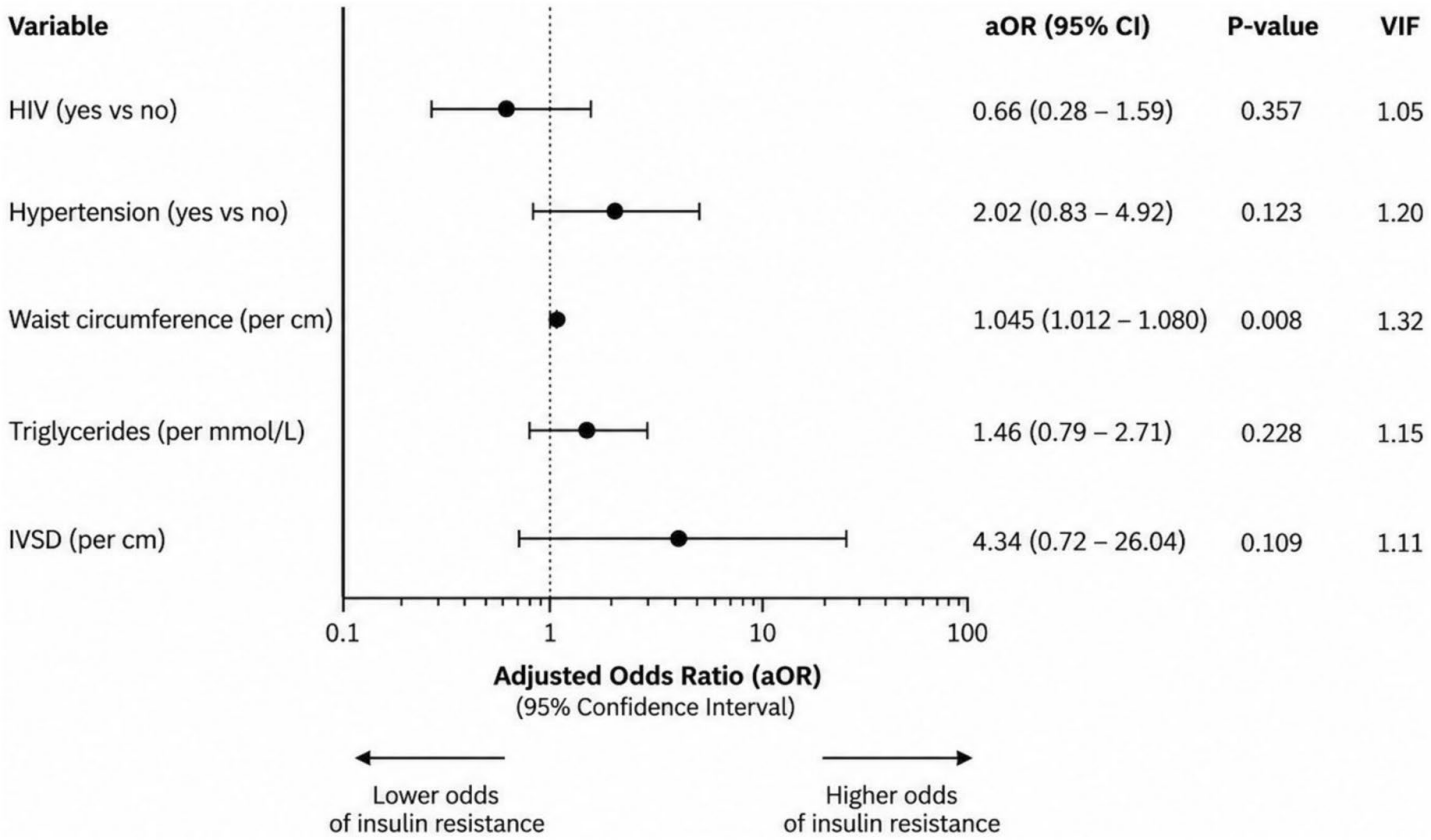
Shows multivariate analysis of factors associated with IR, where various predictors association to the outcome was tested.

## 4 DISCUSSION

### 4.1 Summary of main findings

In this cross‐sectional study from a high HIV‐burden setting, we identified a 15.9% prevalence of IR. The key correlates of IR were traditional cardiometabolic risk factors: central obesity (elevated waist circumference). Notably, after multivariate adjustment, HIV serostatus itself was not an independent determinant of IR in this cohort.

The lack of an independent association between HIV status and IR in our adjusted model is intriguing. It may reflect the virologically suppressed and clinically stable nature of PLWH, where the cardiometabolic risk profile is increasingly dominated by traditional factors like obesity and hypertension rather than HIV‐specific factors such as ART or residual inflammation (Wang et al., 2025). These results reflect findings from a national survey conducted in South Africa that revealed that PLWH were more susceptible to developing hypertension and metabolic syndrome in comparison to the HIV negative group (Magodoro et al., 2022).

In further demonstration of our study findings, an association of waist circumference with IR reinforces the central role of visceral adiposity in the pathogenesis of metabolic dysfunction. Visceral fat is metabolically active and contributes disproportionately to IR through increased release of free fatty acids into the portal circulation, promoting hepatic lipid accumulation and impaired insulin signaling (Chait & den Hartigh, 2020; Tchernof & Després, 2013). This lipotoxic milieu interferes with insulin receptor substrate phosphorylation and downstream Phosphatidylinositol 3‐Kinase/Protein Kinase B (PI3K‐Akt) signaling (Lipke et al., 2022; Zhao et al., 2023), a mechanism demonstrated in Lan et al.'s experimental study (Lan et al., 2025).

Visceral adiposity is particularly relevant to the metabolic health of people living with HIV, in whom long‐term ART, aging, changes in body composition, and persistent immune activation may contribute to an adverse cardiometabolic profile (Asgedom et al., 2024). This is of particular relevance in sub‐Saharan Africa, where metabolic syndrome and its components are increasingly recognized among PLWH receiving ART. In Zambia, metabolic syndrome has similarly been reported among ART‐experienced adults, with associations involving anthropometric measures, including waist circumference (Hamooya et al., 2021). Thus, the elevated waist circumference observed among participants with IR in our study may represent part of a broader cardiometabolic phenotype that is increasingly relevant to long‐term HIV care.

### 4.2 Strengths and limitations

This study is among the few in sub‐Saharan Africa to comprehensively assess the relationship between IR and cardiovascular risk in PLWH using an integrated cardiometabolic analysis. The inclusion of both PLWH and PLWTH strengthened comparative analysis, while standardized methods and validated tools, including echocardiographic, vascular, and biochemical assessments, enhanced data reliability.

However, the cross‐sectional design limits causal inference, and the modest sample size may have reduced statistical power for some associations. Reliance on surrogate IR measures (HOMA‐IR) and the single‐center setting may affect generalizability. Furthermore, unmeasured factors such as diet, physical activity, psychosocial stress, and specific ART regimens could be important confounders. Despite these limitations, the study provides important insights into cardiometabolic risk mechanisms.

## 5 CONCLUSION

In a Zambian cohort with a high prevalence of HIV, IR was predominantly associated with a cluster of traditional cardiometabolic risk factors, central obesity. HIV status was not an independent correlate after accounting for these factors. These findings argue for a shift addressing metabolic disease in Africa towards integrated, routine screening and aggressive management of central obesity to mitigate the growing burden of IR and its cardiovascular sequelae.

## AUTHOR CONTRIBUTIONS

S.L., B.M.H., and S.K.M. conceptualized the study, and S.L. wrote the draft manuscript. S.L., B.H., H.H., M.C., P.C.L., L.M., J.P.P., F.M.C., M.S., A.K., B.M.H., and S.K.M. edited and reviewed different sections of the manuscript. S.L., M.S., and S.K.M. conceptualized the framework and finalized the manuscript. S.K.M. and B.M.H. supervised the work. All authors contributed to the article reviews, edited, and approved the final version of this manuscript.

## FUNDING INFORMATION

This work was supported by the Fogarty International Center and National Institute of Diabetes and Digestive and Kidney Diseases of the National Institutes of Health grants R21TW012635 (S.K.M. and A.K.), R01HL147818 and R01HL144941 (A.K.), National Center for Advancing Translational Sciences grant UL1 TR002243, the Tennessee Center for AIDS Research grant P30 AI110527, and National Heart, Lung, and Blood Institute grant K01HL130497 and the American Heart Association Award Number 24IVPHA1297559 (S.K.M. and A.K.).

## CONFLICT OF INTEREST STATEMENT

The authors have declared that no competing interests exist.

## ETHICS STATEMENT

This study was approved on 17th March, 2025, by the Mulungushi University Ethics Review Committee (SMHS‐MU1‐2025‐39). The authors had no access to information that could identify individual participants during or after data collection. The conduct of the research followed the ethical principles outlined in the Declaration of Helsinki.

## CONSENT

All participants provided written informed consent. All data were handled with strict adherence to privacy and confidentiality standards, with appropriate de‐identification applied to protect participant identity.

## Data Availability

Data is available by reasonable request from the corresponding author.
